# High-Throughput Miniaturized 16S rRNA Amplicon Library Preparation Reduces Costs while Preserving Microbiome Integrity

**DOI:** 10.1128/mSystems.00166-18

**Published:** 2018-11-06

**Authors:** Jeremiah J. Minich, Greg Humphrey, Rodolfo A. S. Benitez, Jon Sanders, Austin Swafford, Eric E. Allen, Rob Knight

**Affiliations:** a Marine Biology Research Division, Scripps Institution of Oceanography, University of California San Diego, La Jolla, California, USA; b Department of Pediatrics, University of California San Diego, La Jolla, California, USA; c Center for Microbiome Innovation, Jacobs School of Engineering, University of California San Diego, La Jolla, California, USA; d Division of Biological Sciences, University of California San Diego, La Jolla, California, USA; e Department of Computer Science and Engineering, University of California San Diego, La Jolla, California, USA; Dalhousie University

**Keywords:** DNA metabarcoding, Illumina MiSeq, NGS, acoustic liquid handler, automation, library preparation, metabarcoding, microbial ecology, microbiome

## Abstract

Next-generation sequencing technologies have enabled many advances across biology, with microbial ecology benefiting primarily through expanded sample sizes. Although the cost of running sequencing instruments has decreased substantially over time, the price of library preparation methods has largely remained unchanged. In this study, we developed a low-cost miniaturized (5-µl volume) high-throughput (384-sample) amplicon library preparation method with the Echo 550 acoustic liquid handler. Our method reduces costs of library preparation to $1.42 per sample, a 58% reduction compared to existing automated methods and a 21-fold reduction from commercial kits, without compromising sequencing success or distorting the microbial community composition analysis. We further validated the optimized method by sampling five body sites from 46 Pacific chub mackerel fish caught across 16 sampling events over seven months from the Scripps Institution of Oceanography pier in La Jolla, CA. Fish microbiome samples were processed with the miniaturized 5-µl reaction volume with 0.2 µl of genomic DNA (gDNA) and the standard 25-µl reaction volume with 1 µl of gDNA. Between the two methods, alpha diversity was highly correlated (*R*_2_ > 0.95), while distances of technical replicates were much lower than within-body-site variation (*P* < 0.0001), further validating the method. The cost savings of implementing the miniaturized library preparation (going from triplicate 25-µl reactions to triplicate 5-µl reactions) are large enough to cover a MiSeq sequencing run for 768 samples while preserving accurate microbiome measurements.

**IMPORTANCE** Reduced costs of sequencing have tremendously impacted the field of microbial ecology, allowing scientists to design more studies with larger sample sizes that often exceed 10,000 samples. Library preparation costs have not kept pace with sequencing prices, although automated liquid handling robots provide a unique opportunity to bridge this gap while also decreasing human error. Here, we take advantage of an acoustic liquid handling robot to develop a high-throughput miniaturized library preparation method of a highly cited and broadly used 16S rRNA gene amplicon reaction. We evaluate the potential negative effects of reducing the PCR volume along with varying the amount of gDNA going into the reaction. Our optimized method reduces sample-processing costs while continuing to generate a high-quality microbiome readout that is indistinguishable from the original method.

## Abstract (precis)

Reduced costs of sequencing have tremendously impacted the field of microbial ecology, allowing scientists to design more studies with larger sample sizes that often exceed 10,000 samples. Library preparation costs have not kept pace with sequencing prices, although automated liquid handling robots provide a unique opportunity to bridge this gap while also decreasing human error. Here, we take advantage of an acoustic liquid handling robot to develop a high-throughput miniaturized library preparation method of a highly cited and broadly used 16S rRNA gene amplicon reaction. We evaluate the potential negative effects of reducing the PCR volume along with varying the amount of gDNA going into the reaction. Our optimized method reduces sample-processing costs while continuing to generate a high-quality microbiome readout that is indistinguishable from the original method.

## INTRODUCTION

Next-generation sequencing prices have steadily decreased over the past decade with the advent of new technologies and improved chemistries of existing sequencing instruments. Library preparation costs, however, have remained the same, leading researchers to develop and test methods to reduce costs by automating assays and reducing reaction volumes for the most expensive library preparation methods, including cDNA synthesis (1) and whole-genome sequencing (2) applications. However, the protocols for the most widely used methods in molecular biology, such as amplicon sequencing, have not been affected by this movement towards miniaturization and automation.

Amplicon sequencing of 16S rRNA, 18S rRNA, internal transcribed spacer (ITS), and other marker genes are commonly used to survey microbial ecosystems, and for small numbers of samples, the effort and upfront cost to reduce reaction volumes have not historically been worthwhile. With the advent of large-scale microbiome sequencing projects with tens of thousands of samples (3, 4), there is now a strong motivation to bring the benefits of miniaturization and automation to this technique. For example, with a 5- to 10-fold reduction in reagent costs as achieved in other reports (1–2), the Earth Microbiome Project (EMP; earthmicrobiome.org), the largest crowd-sourced microbiome project (3), would have saved $500,000 to $1,000,000 in library preparation costs alone, enough to cover the entire sequencing costs for the project. Thus, optimizing this method to reduce costs and increase throughput will be of great utility to many researchers.

Building on the globally recognized protocols for 16S rRNA sequencing adopted by hundreds of labs in the EMP, the purpose of our study was to develop a low-cost 384-sample miniaturized amplicon library preparation protocol and demonstrate its utility using the EMP 16S rRNA V4 primers. We evaluated the effects of reducing PCR volumes and varying input genomic DNA (gDNA) volume on the microbiome signatures, as measured by alpha and beta diversity using four microbial communities of varied diversity and complexity. We then demonstrate that the optimized miniaturized 5-µl-volume protocol performs as well as the standard 25-µl-volume protocol on a diverse set of fish mucus samples.

## RESULTS

### Validation of miniaturized library preparation method.

Existing methods for automating amplicon-based library preparation methods using the EpMotion robots have brought down consumable and reagents costs to $3.44 per sample when scaling to 384 samples. To determine if cost savings could be realized by miniaturizing the PCR volume and gDNA input, we evaluated the quality and reproducibility of assessing microbial communities at the reduced reaction volumes compared to full-scale reactions. We compared the following four common microbial communities of increasing diversity: molecular-grade water (negative control), the commercially available Zymo mock community (ZMC), seawater, and marine sediment in four replicates across eight different input volumes of gDNA. Success rates did not vary across PCR volumes when rarified to 1,000 reads, indicating that miniaturizing the reaction did not cause issues of insufficient materials or reactants to obtain quality libraries for sequencing. Intriguingly, when we applied a higher read threshold by rarefying at 10,000 reads, success was significantly improved with lower PCR volumes of 5 µl from 21% to 46% (*P* < 0.05) and further improved to 69% at a 2.5-µl reaction volume (*P* < 0.001) (see Fig. S2a and b in the supplemental material). Decreasing the PCR volumes did not affect alpha (Fig. 2a) or beta (Fig. 2b) diversity (*P* > 0.05, Kruskal-Wallis).

Decreases in within-sample alpha diversity (in complex samples) and increases in between-sample beta diversity occurred when gDNA input volumes were less than 0.05 µl, with the most drastic effects at 0.005 µl; we thus opted for gDNA input of 0.2 µl (Fig. 2c and d). Sample volume-related changes could be due to subsampling effects or background contamination. Accurate and reproducible composition of the positive-control samples (ZMC) was obtained across all PCR volumes and with gDNA volumes over 0.005 µl (Fig. 3).

### Application of method to diverse environmental sample set.

To further validate the miniaturized library preparation method across diverse sample types, we sequenced microbiome samples from five body sites of 46 individuals of the marine fish Scomber japonicus (Pacific chub mackerel) across seven months of sampling (Fig. 4a). Sample exclusion testing indicated a read cutoff of 1,362 reads (Fig. S3). Alpha diversity was highly correlated when samples were processed through the two different methods, for richness and Shannon diversity, respectively (*P* < 0.0001, *r*^2^ = 0.9534; *P* < 0.0001, *r*^2^ = 0.962) (Fig. 4b and c). Interestingly, the slopes for both richness and Shannon diversity indicate a slightly higher alpha-diversity estimate for samples processed with the EpMotion than with the Echo 550. Both weighted (Fig. 4d) and unweighted (Fig. 4e) UniFrac distances for technical variation (PCR methods) across all body sites with enough replicates were significantly smaller than the total variation within a body site (Mann-Whitney, *P* < 0.0001).

## DISCUSSION

### Financial and reproducibility implications for a miniaturized amplicon library preparation.

Automation is revolutionizing sample acquisition, sample processing, and data analysis in many fields of science. While sequencing costs have dramatically decreased over time, the cost of commercial next-generation sequencing (NGS) library preparation has largely remained constant, at around $10 to 20 per sample.

**FIG 1:**
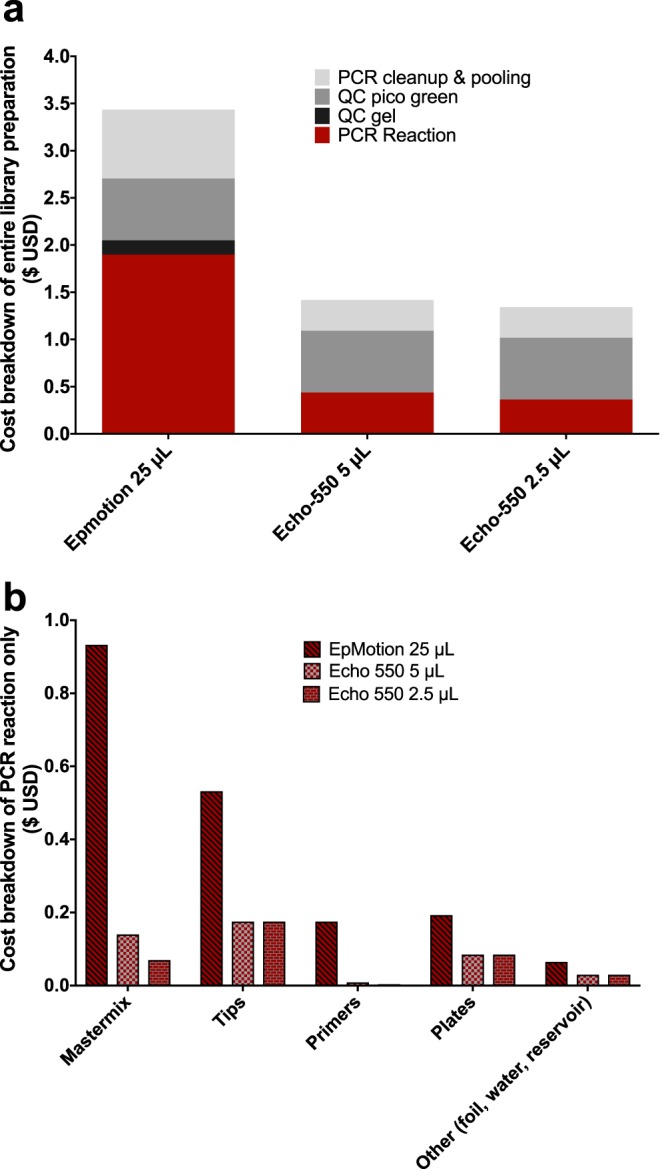
Miniaturized amplicon library preparation reaction reduces costs while maintaining microbiome readout integrity. (a) Total reagent and consumable costs for library preparation across three reaction volumes, 25 µl, 5 µl, and 2.5 µl, which includes the setup of the PCR along with library QC and pooling. (b) The breakdown of costs associated with the PCR (red section from 1a) are further expanded.

We were able to reduce costs by an additional 58% to $1.42 per sample when using a miniaturized 5-µl reaction volume or $1.34 per sample with a 2.5-µl reaction volume (Fig. 1a). Since the cost reduction for going from 5 µl to 2.5 µl is minimal, and concerns for evaporation increase with lower volumes, we consider the 5-µl reaction volume to be the optimal compromise. The costs savings are primarily a result of a decrease in use of PCR mastermix ($0.933 for 25 µl versus $0.14 for 5 µl) and tips ($0.532 for 25 µl versus $0.175 for 5 µl) (Fig. 1b), which when scaling to 50,000 samples a year can result in annual savings of $17,847 and $39,655, respectively (Fig. S1a and b). Other significant cost savings when miniaturizing the reaction include primer costs ($0.166 per sample), plate consumables ($0.108 per sample), PCR cleanup and pooling ($0.406 per sample), and gel quality control (QC) ($0.152 per sample) (Fig. 1). In full, a total cost reduction of $1.02 from miniaturizing the library preparation 5-fold results in an annual cost savings of $101,000 for 50,000 samples and $202,000 for the 100,000 samples typical of our laboratory (Fig. S1c). Since it is common to multiplex 768 samples on a single Illumina sequencing lane, we calculated the library preparation cost savings per run to be $1,549, or slightly more than a MiSeq 2 × 150-bp sequencing run according to current pricing available on the Illumina website (as of August 2018).

**FIG 2:**
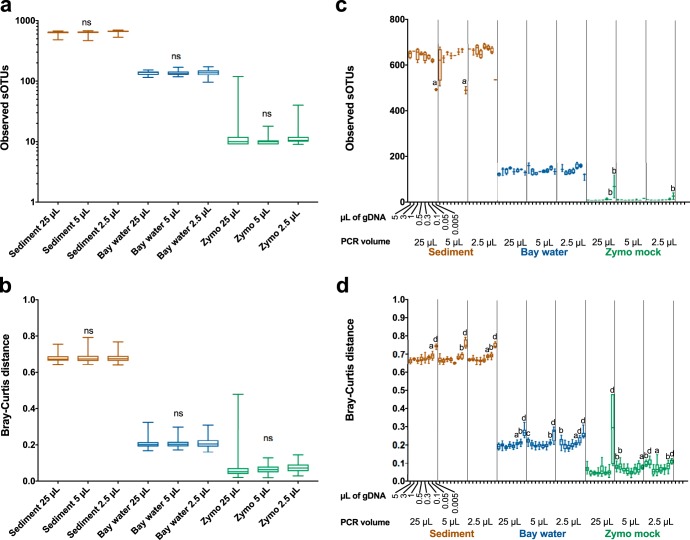
Measured microbial effects of altering PCR volume and DNA input volume. The effects of PCR volumes on alpha diversity through richness (a) and beta diversity through Bray-Curtis distances (b) within sample types measured by nonparametric Kruskal-Wallis test. The effects of gDNA input (5 µl, 3 µl, 1 µl, 0.5 µl, 0.3 µl, 0.1 µl, 0.05 µl, or 0.005 µl) on alpha diversity through richness (c) and beta diversity through Bray-Curtis distances (d) within sample types with significance calculated by comparing sample replicates across gDNA volumes to control (25-µl reaction volume, 1 µl gDNA) using nonparametric Kruskal-Wallis, Benjamini-Hochberg false-discovery rate (FDR) (panel a, *P* < 0.05; panel b, *P* < 0.01; panel c, *P* < 0.001; panel d, *P* < 0.0001). All samples were rarified to 1,000 reads. All error bars indicate range (minimum to maximum) to show the absolute variability. ns, nonsignificant.

**FIG 3:**
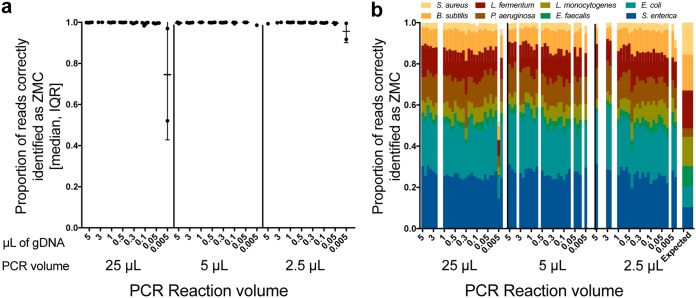
Validation of Zymo mock community (ZMC) control samples rarified to 1,000 reads. (a and b) Total relative abundance of reads (a) and the compositional breakdown from control samples which were classified as one of the eight bacteria (b) across PCR volume and gDNA input volume. White areas in panel b indicate samples that did not have at least 1,000 reads and thus were omitted from the analysis. IQR, interquartile range.

**FIG 4:**
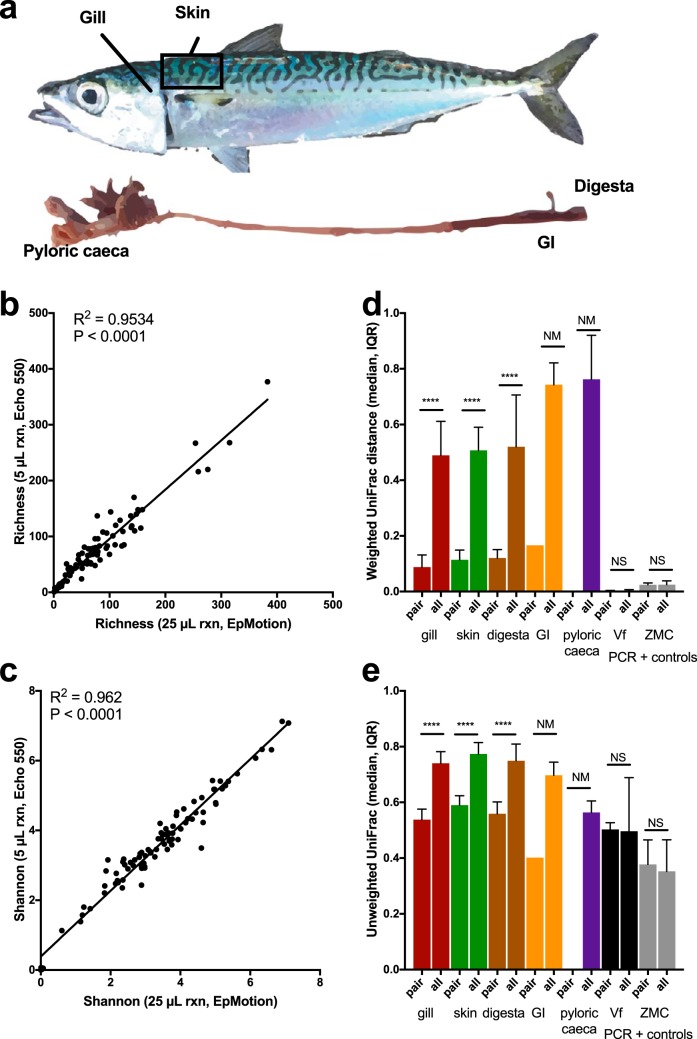
Validation of miniaturized 16S rRNA PCR method across diverse sample set, with an assessment of technical variation. (a) Five mucosal sites, including gill, skin, digesta, gastrointestinal tract, and pyloric caeca were sampled from 46 *Scomber japonicus* mackerels across seven months in 2017. Vf, Vibrio fischeri isolate; ZMC, Zymo mock community. Both are considered PCR positive controls since the compositions were known prior to sequencing. Correlation of alpha diversity as measured by sOTU richness (b) and Shannon entropy (c) comparing samples processed through the standard EpMotion 3× 25 µl PCR with 1 µl of gDNA and the miniaturized Echo 3 × 5 µl PCR with 0.2 µl of gDNA (Spearman). Assessment of differences in beta-diversity distances of weighted UniFrac (d) and unweighted UniFrac (e) variation of within-sample distances (comparing same sample processed through 25-µl versus 5-µl reaction [rxn] volume) versus within sample type variation. Statistical significance was determined by a pairwise nonparametric Mann-Whitney (two-tailed) test comparing distances in technical variation to distance variations within body site for each body site individually. Significance indicated by ****, *P* < 0.0001; NS, nonsignificance, *P* > 0.0001. Where sample size was not large enough, comparisons are indicated by NM, not measured.

10.1128/mSystems.00166-18.1 FIG S1 Cost savings due to switching from original 3× PCR 25-µl volume library preparation on the EpMotion to 3× PCR 5-µl volume library preparation on the Echo-550 acoustic liquid handler. Reagent and consumable cost projections in dollars of the two methods for scaling to larger throughput for tips (a), PCR mastermix (b), and the complete library preparation (c). Download FIG S1, EPS file, 0.1 MB. Copyright © 2018 Minich et al. 2018 Minich et al. This content is distributed under the terms of the Creative Commons Attribution 4.0 International license.

10.1128/mSystems.00166-18.2 FIG S2 Effects of PCR volume on sequencing success rarified at 1,000 reads (a) and 10,000 reads (b). Columns represent median and interquartile range of percent success of all three environments (sediment, seawater, and Zymo mock community) binned per library preparation PCR volume. Columns were compared using one-way ANOVA with Tukey’s *post hoc* test (****, *P* < 0.0001; ***, *P* < 0.001; **, *P* < 0.01; *, *P* < 0.05). Download FIG S2, EPS file, 0.1 MB. Copyright © 2018 Minich et al. 2018 Minich et al. This content is distributed under the terms of the Creative Commons Attribution 4.0 International license.

10.1128/mSystems.00166-18.3 FIG S3 Limit of detection titration curves generated from controls to determine sample exclusion criteria of 1,362 reads. The *x* axis depicts the total number of reads per sample after Deblur sOTU filtering, whereas the *y* axis indicates the proportion of reads correctly identified in the controls as either *Vibrio fischeri* or ZMC. The allosteric sigmoidal equation is used to determine the value (read number) at which 0.9 proportion of the sample is assigned correctly. Download FIG S3, EPS file, 0.2 MB. Copyright © 2018 Minich et al. 2018 Minich et al. This content is distributed under the terms of the Creative Commons Attribution 4.0 International license.

Our results demonstrate that the miniaturized PCR protocol maintains data quality with respect to traditional methods while achieving substantial cost advantages (more than 10-fold relative to commercial kits) and producing much less plastic tip waste. The use of an acoustic liquid handler also permitted dramatic decreases in sample volume usage, which may be important when using precious historical samples (5) with limited volumes or running large numbers of assays per sample. While we performed this miniaturization using an instrument produced by Labcyte (San Jose, CA, USA), similar results may be achievable with the instruments from EDC Biosystems (Fremont, CA, USA), although we have not evaluated performance on the EDC Biosystems instrument. Likewise, other nanoliter-dispensing robots would be able to achieve similar volume miniaturization but not obtain the significant environmental and cost savings from the elimination of tips. Overall sample success may actually be improved when performing library preparation reactions at lower volumes. Higher success at lower volumes could be explained by higher proportions of amplicon going into the library pool or improved efficiency of enzymatic reactions at lower volumes (6–8). Alpha- and beta-diversity measures were highly correlated across methods. While we did not explicitly test well-to-well contamination, other studies have demonstrated that contamination may be lower when using acoustic liquid handlers over traditional pipette-based liquid handlers for molecular assays while also having higher dispensing accuracy (9). Finally, while we demonstrate the utility of this method for the 16S rRNA gene, this method could easily be applied to any amplicon based library preparation method. We therefore recommend the new protocol for large-scale studies.

Although the acoustic liquid handling instrument on which it relies is expensive, considerable cost savings can be achieved, particularly in the context of a core facility or in a high-throughput laboratory. Further advances in low-volume liquid handling will be expected to make this technology available to more individual research groups. The ability to perform amplicon sequencing in much higher throughput makes accessible large-scale ecological and epidemiological applications that are presently cost-prohibitive.

## MATERIALS AND METHODS

### Sample collection.

Four sample types of varied microbial diversity were chosen to first optimize and compare sequencing success. These samples included a marine sediment sample from the Scripps Institution of Oceanography (SIO) pier, filtered sea water from the San Diego bay, the ZymoBIOMICS DNA mock community (ZMC) (catalog no. D6305; Zymo Research), and molecular-grade water (catalog no. W4502; Sigma-Aldrich) as a negative control. To collect samples representing true ecological diversity, 46 Scomber japonicus mackerels were collected across 16 sampling events from the end of the SIO pier (32.867, −117.257) from 28 January 2017 to 4 August 2018. Fish were caught using hook and line at sunset, euthanized, and then immediately wrapped in foil and stored on dry ice and then −80°C prior to dissection. Upon dissection, ∼50 mg of hindgut and pyloric caeca was placed into extraction tubes while the gill, skin, and digesta were swabbed (catalog no. 806-wc; Puritan) for processing by DNA extraction.

### DNA extraction.

Samples were processed using the standard 16S rRNA Earth Microbiome Protocol (earthmicrobiome.org). Specifically, gDNA was extracted using single tubes, followed by DNA magnetic bead cleanup with the Mo Bio PowerMag kit (catalog no. 27000-4-KF), which improves the limits of detection for low-biomass samples (10). Additional positive and negative DNA controls were included so that sample exclusions based on read counts could be calculated (10).

### 16S rRNA gene amplicon library preparation.

Extracted gDNA was then PCR amplified in triplicate reactions for 35 cycles using the EMP 16S V4 515f/806rB barcoded primers (11, 12) at a 25-µl (*n* = 32) volume or miniaturized 5-µl (*n* = 32) or 2.5-µl (*n* = 32) reaction volumes with eight volumes of gDNA (5 µl, 3 µl, 1 µl, 0.5 µl, 0.3 µl, 0.1 µl, 0.05 µl, and 0.005 µl) in quadruplicate from the four communities (negative controls, ZMC, seawater, and sediment) normalized to 3 ng ul^−1^ DNA. Multiple body sites, including the gill, skin, digesta, gastrointestinal (GI) tract, and pyloric caeca from 46 S. japonicus individuals spanning seven months of sampling were amplified in standard 25-µl-volume PCR setup with 1 µl gDNA input using the EpMotion 5075 versus the optimized 5-µl PCR setup using the Echo 550 and 200 nl gDNA input. Positive gDNA controls of Vibrio fischeri and ZMC were also included.

**Sample sequencing and data processing.** Amplicons were quantified using a PicoGreen assay (Thermo Fisher Scientific, San Diego, CA), and then 2 µl of each sample amplicon library was equally pooled into a single tube and then sequenced on a MiSeq 2 × 150-bp v2 kit. Raw sequencing files were uploaded and processed with Qiita (qiita.ucsd.edu) and QIIME 1.9.1 (13), with the first read trimmed to 150 bp and processed through Deblur (14), a *de novo* sOTU picking method. A phylogenetic tree of the 16S sub-operational taxonomic unit (sOTU) single-sequence tags was created using SEPP (15). The minimum rarefaction depth for experiment 1 was 1,000 reads to exclude most negative controls (Fig. S4), whereas the depths for experiment 2 were empirically determined by calculating the read counts at which 90% of the reads from the DNA extraction positive controls map back to the positive controls (10). Alpha diversity was calculated using measures of richness and Shannon entropy (16), while beta diversity was calculated using either Bray-Curtis (17) or weighted and unweighted UniFrac (18, 19) distance and visualized in EMPeror (20). Alpha- and beta-diversity statistical significance was tested using a Kruskal-Wallis test (21) and Mann-Whitney test (22). Correlations of alpha diversity between the two methods of the fish samples was performed using Spearman’s rho correlation (23, 24).

10.1128/mSystems.00166-18.4 FIG S4 Read counts per sample type (brown, sediment; blue, seawater; green, ZMC; grey, molecular-grade water nontemplate control). Download FIG S4, EPS file, 0.2 MB. Copyright © 2018 Minich et al. 2018 Minich et al. This content is distributed under the terms of the Creative Commons Attribution 4.0 International license.

**Data availability.** All protocols associated with the Echo-550 scripts have been uploaded and are publicly available on protocols.io. The links can be found here: https://www.protocols.io/view/high-throughput-miniaturized-16s-rrna-amplicon-lib-u2ieyce. All raw and processed 16 amplicon sequencing data and metadata from the first PCR validation experiment (Qiita study identification [ID] 11432; https://qiita.ucsd.edu/study/description/11432; EBI study accession no. ERP109772) and the second validation experiment, including the full fish mucosal time series data set (Qiita study ID 11721; https://qiita.ucsd.edu/study/description/11721; EBI study accession no. ERP109537), are publicly available in Qiita and EBI.
